# Achieving Parity of Esteem of Mental Healthcare in the UK

**DOI:** 10.1192/j.eurpsy.2022.1620

**Published:** 2022-09-01

**Authors:** Y. Sheikh, D. Gakhar, M.-H. Asunramu, H. Low, H. Yassin, K. Muthukumar

**Affiliations:** 1 King’s College London, Faculty Of Medicine & Life Sciences, London, United Kingdom; 2 Imperial College London, Imperial College Business School, London, United Kingdom

**Keywords:** Parity of esteem, Mental Health Policy, mental health, United Kingdom

## Abstract

**Introduction:**

Mental illness is the single largest cause of disability in the UK, with one in four individuals suffering from a mental health problem. Despite this, only 13% of the NHS budget goes towards treatment of mental illness. It is thus unsurprising that addressing the parity of esteem of mental health has been highlighted as a major priority for the healthcare system, with the NHS Five Year Forward plan aiming to achieve this by 2020.

**Objectives:**

To explore the barriers to achieving parity of esteem of mental healthcare in the UK and develop recommendations for implementation.

**Methods:**

Narrative review of literature and synthesis of findings

**Results:**

Three key barriers to achieving parity of esteem of mental health were identified: the current mental health investment standard (MHIS), medical sub-specialisation, and access to acute day units (ADU). The following recommendations were thus synthesised: to increase the time-period to measure MHIS increments, integrating mental health teaching into specialty training programmes, and increasing the availbility of ADUs to crisis referral teams.

**Conclusions:**

Addressing the disparity between mental and physical health is a major priority for the NHS. This research provides an overview of current barriers and suggests recommendations for improvement. By prioritizing improvement in the MHIS, integrating mental health teaching into specialty training, and increasing access to ADUs, the NHS formulates an excellent founding to achieving the ultimate goal, parity of esteem of mental health.

**Disclosure:**

No significant relationships.

